# Gendered Vice Complaints: 911 Calls Reporting Sex Work in Chicago Neighborhoods, 2017-2020

**DOI:** 10.1177/15570851241284404

**Published:** 2024-09-19

**Authors:** Lexi Harari, Chris M. Smith, Taylor Domingos, Sharon S. Oselin, Emily Hammond

**Affiliations:** 1 Presley Center of Crime & Justice Studies, University of California, Riverside, CA, USA; 2 Department of Sociology, 7938 University of Toronto, Toronto, ON, Canada; 3 Department of Sociology, 3270 Northwestern University, Evanston, IL, USA; 4 Department of Public Policy and Sociology, University of California Riverside, Riverside, CA, USA

**Keywords:** sex work, arrests, 911 calls, neighborhood disorder, urban social control

## Abstract

Urban communities use 911 to demand “quality-of-life” police responses and control neighborhood “disorder.” Less is known about the neighborhoods calling 911 to report sex work perceived as disorderly. We investigate the frequency and spatiality of 911 calls reporting sex work across Chicago census tracts relative to arrests and model the relationships between these and neighborhood characteristics. We find that 911 calls spread across Chicago with moderate clustering, but the highest social control of sex work occurs in the West Side. Increased 911 calls come from gentrifying, commercial, and Black neighborhoods, but socioeconomic disadvantage has the largest increase on vice complaints.

## Introduction

Urban residents and community members often take proactive approaches—such as calling 911—to resolve “quality-of-life” issues that arise in their neighborhoods. Indeed, most 911 calls focus on quality-of-life concerns around “disorder” (e.g., witnessing substance use, noise complaints, loitering) (Neusteter et al., 2020). People use 911 to report these types of incidents with the belief that street level disorderly behaviors can generate more serious criminal activity within their neighborhoods (Beckett & Herbert, 2010; Skogan, 1990), rendering a police response a warranted solution. In turn, police officers spend a significant portion of their time responding to 911 calls and the complaints of disorder that fuel them (Herring, 2019; Laniyonu, 2017).

While the quality-of-life policing scholarship often considers forms of disorder like homelessness, loitering, or noise complaints (Herring, 2019; Laniyonu, 2017), other disturbances to quality-of-life can include behaviors associated with sex work (Skogan, 1990). Criminalized sex markets are subjected to substantial formal and informal social controls, especially when operating in outdoor public spaces (Dewey & St. Germain, 2015; Kingston, 2014). Although 911 callers and police officers are unlikely to witness the actual exchange of criminalized sex services for money, suspicions about these transactions spur much scrutiny. Certain behaviors can be interpreted as signs of sex work: standing in particular places for extended periods of time, flagging down cars, wearing revealing clothing, and numerous fleeting interactions between individuals. Unlike other forms of neighborhood disorder that generate anger or fear of crime, visible sex work activities generate moral indignation that results in 911 calls to summon police (Skogan, 1990). Indeed, calling 911 is a common and powerful tool used by civilians, business owners, and community members to displace and eliminate such vice activities; however, less is known about how vice complaints distribute across cities and whether they vary by neighborhood.

The unique dimensions of the illicit sex market and efforts to control it are especially relevant to feminist criminology given the gender composition of sex workers and the moral evaluations that fuel many complaints against them. Sex work is the only criminalized victimless activity that disproportionately includes and targets transgender and cisgender women providers (Becker & McCorkel, 2011; Britton, 2000; Vanwesenbeeck, 2013). Thus, illicit sex work provides a unique window into research on 911 calls as a tool of urban social control. Our goal is to build on the urban social control of disorder and quality-of-life policing scholarship through a focus on illicit sex work that has had fewer quantitative investigations. Doing so will clarify if certain neighborhoods disproportionately use 911 calls to report sex work and will potentially contribute a more gendered spatial dimension to the existing literature. By examining the reporting of a gendered, victimless, and economic crime, for which 911 calls are more likely to imply moral judgments associated with a sense of disorder (Shdaimah et al., 2014; Skogan, 1990), our investigation taps into criminalized dimensions of gender and sexuality.

We use Chicago data to examine the distribution of 911 calls reporting sex work across gentrifying, commercial, and disadvantaged neighborhoods from 2017 through 2020. Specifically, we analyze the neighborhood concentration of 911 calls reporting sex work relative to their arrests, and we test the relationships of neighborhood characteristics on sex work 911 calls and arrests. Our findings reveal that 911 calls spread across Chicago with only moderate spatial clustering, but the highest social control of sex work occurs in the West Side. Also, increased 911 calls come from gentrifying, commercial, and Black neighborhoods, but socioeconomic disadvantage has the largest increase on vice complaints as well as arrests.

## 911 Calls and Neighborhoods: Urban Social Control of Disorder

When police officers and civilians interact, their contact often begins with a 911 call (NENA, 2022). Approximately 240 million 911 calls are made annually in the US, and most of these calls are not for serious crimes in action (NENA, 2022; Neusteter et al., 2020). Instead, these calls often task police with responding to quality-of-life concerns and addressing public disorder, particularly visible forms that urban residents perceive as nuisances such as substance use, noise, or loitering (Laniyonu, 2017; Skogan, 1990). In a study of nine major US police departments, disorder calls made up 16% of 911 calls, second only to traffic-related incidents (Lum et al., 2022). Calls provide an efficient method for civilians to request support to resolve disorder and associated quality-of-life issues, and police spend a significant portion of their time responding to these requests (Neusteter et al., 2020).

The urban social control of disorder requires an uneven partnership between the public and the police. Local residents and community members define disorder as violations of their presumably shared values and demand police responses to their complaints of disorder (Herring, 2019; Skogan, 1990). Policing disorder, however, is seldom compatible with modern policing evaluations and crime control goals, as police are limited in their capacity and motivation to manage it (Herring, 2019; Skogan, 1990). Scholars have examined the dynamics of the urban social control of disorder through complaint-oriented policing (Herring, 2019), displacement through spatial exclusion laws and ordinances (Beckett & Herbert, 2010), and postindustrial order-maintenance policing (Golash-Boza et al., 2023; Laniyonu, 2017; Sharp, 2014). While civilian complaints activate these policing types through 911 calls, not all urban neighborhoods experience disorder or complaints to control them equally. The social control of disorder varies by neighborhood characteristics with different motivations for doing so.

### Gentrifying Neighborhoods

Gentrifying neighborhoods use 911 to demand quality-of-life policing and control neighborhood disorder (Beck, 2020; Doering, 2020; Fagan & MacDonald, 2013; Laniyonu, 2017). Gentrifiers rely on police services to ensure that their capital investment in their new neighborhoods will not be interrupted by disorderly persons perceived as threats to urban growth and ballooning property values (Beck, 2020). Campaigns to rid gentrifying neighborhoods of disorder have focused on removing gangs, drug dealers, and sex workers (Doering, 2020; Gordon, 2022; Lyons et al., 2017). Neighborhood watch groups form, vocal residents participate in police community meetings, and gentrifiers activate 911 call trees to flood operators with complaints about certain properties (Doering, 2020). In many American cities, these gentrifiers’ campaigns have racialized targets and consequences as police respond to gentrifying neighborhoods’ complaints with increased quality-of-life policing and racial boundary maintenance (Golash-Boza et al., 2023; Gordon, 2022).

### Commercial Neighborhoods

People in commercial districts also take concerted action to prevent crime and control disorder by placing 911 calls (Garland, 2001; Herring, 2019). Business owners and workers rely on police services to eliminate illicit activities and control the spaces surrounding their storefronts, such as reporting traffic congestion related to sex solicitation or people sleeping near business entrances (Herring, 2019; Lyons et al., 2017). Ambient populations (local residents, within-metropolitan commuters, and tourists) also contribute to 911 calls in commercial neighborhoods, as the density of ambient populations impacts crime rates (Tucker et al., 2021). This could deter crime as ambient populations serve as guardians willing to intervene and take actions to prevent it (Taylor, 1988). This indicates that potentially high numbers of actors (presumably with cell phones) present in commercial areas use 911 to report disorder. For example, Minkoff (2016) found an increase in 311 service requests in commercial districts in New York City where there are more feet on the sidewalks and vehicles on the streets.

### Socioeconomically Disadvantaged Neighborhoods

While 911 calls are frequent across most communities, they occur at higher rates in socioeconomically disadvantaged neighborhoods, accounting for the most common type of police-citizen interaction in these settings (Hagan et al., 2018). Rather than the quality-of-life concerns driving policing in gentrifying neighborhoods, police respond to poor, high crime neighborhoods through an orientation of crime control and overpolicing even when residents complain of disorder (Gordon, 2022). US urban legacies of racial segregation compound with neighborhood socioeconomic disadvantage, resulting in poor Black neighborhoods disproportionately experiencing high crime rates and intensive police activity (Peterson & Krivo, 2010). Moreover, police are slow to respond to service calls in poor, Black, high crime neighborhoods, generating the “overpolicing-underpolicing paradox” whereby calls for police help are ignored but unwanted police encounters among young, Black and Brown men are regular routines (Jones, 2014; Rios, 2011).

These tensions result in high degrees of cynicism and distrust in the police (Bell, 2016; Hagan et al., 2022). Residents harbor long-lasting cynicism and hostility, but, given few other options, they reluctantly acknowledge that police services are the only way to achieve any order or protection (Carr et al., 2007; Hagan et al., 2022). Cynicism and distrust operate at the neighborhood level as residents collectively endorse the belief that police are unprepared and/or inefficient at protecting their communities (Oliveira & Jackson, 2021; Tyler, 2004). This collective understanding typically stems from direct or vicarious experiences of negative interactions with the police, which disproportionately affect racial/ethnic minorities and socioeconomically disadvantaged residents (Hagan et al., 2022; Weitzer & Tuch, 2006). Rather than police responding directly to quality-of-life 911 calls in disadvantaged neighborhoods, they respond with broad crime control tactics that disproportionately affect the urban poor, fortifying existing racial, gender, and class inequalities within cities and in particular neighborhoods (Beckett & Herbert, 2010; Gordon, 2022; Rosentel et al., 2021).

## Gender and Street-Based Sex Work

Existing research concludes that cisgender men are the predominant purchasers of sex, while cisgender and transgender women comprise the majority of sex providers (Jacobs & Miller, 1998; Vanwesenbeeck, 2013). Accordingly, sex work is the only victimless economic crime that disproportionately includes and targets women (Becker & McCorkel, 2011; Britton, 2000). At the intersection of class, race/ethnicity, and gender, most sex work scholars find that street-based workers are disproportionately poor women of color participating in the illicit sex economy for their subsistence (Maher, 1997; Oselin, 2014; Venkatesh, 2006). Work is one of the most important sites where gender is performed, displayed, and interpreted (West & Zimmerman, 1987); thus, sex work is one of the more “feminized” occupations (Benoit et al., 2015).

As both a criminalized and stigmatized occupation, street-based sex work has long been subjected to a high degree of social control from outsiders and the criminal justice system. Community members, residents, and business owners frequently draw on gendered stereotypes and performance cues to make determinations about who constitutes sex workers based upon their dress (e.g., revealing clothing, high-heeled shoes), behaviors (e.g., waving at cars, talking with drivers), and physical locations (e.g., walking or congregating in known strolls) (Oselin et al., 2022; Skogan, 1990). By contrast, men who sell sex are often ignored or presumed not to be sex workers, due to gendered stereotypes and their concerted efforts to distance themselves from this feminized occupation (Oselin, 2018). Shdaimah and colleagues’ (2014) study on perceptions of outdoor sex workers reveals community members’ assumptions that providers are women and clients are men, driving this form of perceived disorder as differently gendered than other forms. Public concerns about sex work include morality judgements but also auxiliary disorder that can accompany it, such as greater noise and trash, street harassment, and the transmission of disease (Minichiello & Scott, 2014; Shdaimah et al., 2014). These concerns have spurred organized community activities intended to dampen and ultimately displace outdoor sex work from neighborhoods (Kingston, 2014; Lyons et al., 2017; Skogan, 1990). In some cases, local businesses even collaborate with community members in their efforts to control sex work activities (Beckett & Herbert, 2010). Women sex workers are the usual suspects and targets for these eradication efforts, meaning that they bear the brunt of public scrutiny and judgment (Krüsi et al., 2016).

Community members typically rely on the police or other criminal justice mechanisms to address sex work. Police departments receive thousands of 911 calls from civilians attempting to divert and eliminate sex workers and their activities from their neighborhoods (Lum et al., 2022). Such complaints typically generate police responses to outdoor sex work, which can include warnings to “move along” to another neighborhood or arrests (Cunningham & Kendall, 2011; Oselin et al., 2022). Like the complaints, it is usually women street-based sex workers who are subjected to these unwanted policing encounters. For example, a recent Chicago study revealed that the gender composition of arrests for prostitution were about 70% women and 30% men (Rosentel et al., 2021; see also City of Chicago Mayor’s Office on Domestic Violence, 2006). However, there is some evidence that, in certain locations, police and courts deprioritize the arrests and criminal charges of outdoor sex workers, replacing these with other types of criminal justice interventions focused on “treatment” (Krüsi et al., 2016; Oselin, 2014; Shdaimah & Leon, 2015).

## Current Study

Above we review two somewhat disparate literatures relevant to this study. Our literature review on 911 calls and neighborhoods focuses on how different types of neighborhoods approach the urban social control of disorder: gentrifiers use 911 to demand quality-of-life policing, commercial districts use 911 to improve business fronts with dense ambient populations making calls, and socioeconomically disadvantaged neighborhoods use 911 to manage disorder in spite of distrust in the police and their crime control responses. However, sex work is seldom mentioned or not a primary focus in most of these neighborhood studies. The implication from this literature to the topic at hand is that when publics perceive sex work as a form of disorder, these types of neighborhoods are also likely to call 911 and mobilize the police to activate these tools of urban social control that target transgender and cisgender women workers.

The literature on street-based sex work establishes how this illicit market, its public perceptions, and the complaints and criminal justice management of it, are differently gendered compared to other forms of disorder. Research on sex workers’ interactions with police and community members has largely been qualitative, focusing on how these entities respond to disorder associated with sex work in specific neighborhoods. While such studies offer valuable insights into these dynamics, there are minimal quantitative studies that examine the spatial differences in community perceptions and police interactions with sex workers across cities’ neighborhoods (but see Shdaimah et al., 2014; Skogan, 1990). Indeed, O’Neill et al. (2008) point out that existing scholarly investigations of responses to sex work do not reveal important contextual neighborhood differences. We know little about how collective actions, such as calling 911 to report sex work, or the relationship between calls and arrests vary across neighborhoods within cities.

Understanding these trends offers pivotal insights into neighborhood characteristics as they affect the degree of criminalization of one of the most vulnerable sex worker populations. Thus, our research questions ask: Where are 911 calls reporting sex work originating in Chicago? What are the relationships between neighborhood characteristics and these gendered vice complaints? Given data limitations, we must assume based on prior research, rather than measure, the gender composition of Chicago’s street-based sex market. In doing so, the aim of this research is to examine 911 call complaints and arrests as forms of urban social control of disorder targeting illicit sex work to elucidate if and how these spatial processes might be differently gendered.

## Data and Measures

Chicago is an ideal case to study public complaints about sex work relative to arrests because of data availability and its size as the third largest city in the US. Our study analyzes 911 calls and arrests of sex work in Chicago neighborhoods from 2017 to 2020. Following scholars who examine tract level variation in 911 or 311 calls (Hagan et al., 2022; Laniyonu, 2017; Minkoff, 2016), our unit of analysis is census tracts that we use to approximate Chicago neighborhoods. The 2020 US Census includes 866 census tracts for Chicago, and we use the *tidycensus* package in R to obtain the 2020 boundaries (Walker & Herman, 2021). We drop three tracts from our analysis that had populations of zero as well as the six tracts surrounding O’Hare airport.^1^ Our final analytic sample is Chicago’s 857 tracts, with a mean population of 3,531 people per tract.

### Sex Work 911 Calls

Our primary outcome variable comes from official records of 911 calls reporting sex work to dispatchers. We obtained Chicago’s 911 calls reporting any “prostitution” and “vice complaint in progress/report” from 2017 to 2020 through a Freedom of Information Act (FOIA) request to Chicago’s Office of Emergency Management & Communications. The Office only maintains 911 call records for four years, which bounded the timeline of our study. Due to the short period of time available for this variable from these data, we summed the four years into a single four-year count. Each call record includes the date and year, address (partially redacted block information), and type of call (prostitution/vice). We were unable to obtain any additional information about the caller or the target of the 911 call. We midpointed the partially redacted blocks to addresses (e.g., converting 44xx W Wilcox St to 4450 W Wilcox St) and ran the addresses through the *ggmap* package in R to obtain the latitude and longitude coordinates (Kahle & Wickham, 2013). We then merged these call coordinates to the 2020 census tract boundaries. On average, there were 16,402 calls reporting sex work per year from 2017 to 2020.^2^ Our decision to sum the four years of calls per census tract for our analysis increases the variation across the tracts and minimizes yearly fluctuations. During the four years, the 857 Chicago census tracts had an average of 76.6 (sd = 127.4) sex work calls, with the tracts’ four-year totals ranging from a minimum of zero to a maximum of 2,109 calls. Table 1 presents the descriptive statistics.

**Table 1.** Descriptive Statistics for Social Control of Sex Work and Neighborhood Characteristics (*n* = 857).

	Top 10 percent calls (*n* = 86)	Top 10 percent arrests (*n* = 86)	Top social control (4%, *n* = 37)	Rest of the city (84%, *n* = 722)
Mean sex work 911 call rate^a^	1316.33***	901.05***	1720.50	149.09
Range sex work 911 call rate^a^	539.03–8583.64	62.72–8583.64	540.99–8583.64	0–531.21
Mean sex work arrest rate	111.56***	125.14***	258.91	0.28
Range sex work arrest rate	0–1880.49	5.77–1880.49	5.90–1880.49	0–5.68
Gentrification (%)^b^	15%	19%	11%	12%
Residential (%)	51%***	55%*	53%	59%
Commercial (%)	10%**	11%	9%	12%
Socioeconomic disadvantage index	1.93***	1.08***	1.60	-0.28
Black (%)	76%***	62%***	74%	28%

*Note.* Two-tailed *t* test results with significant mean differences between the top 10% samples and the rest of the city denoted by *** (*p* < .001), ** (*p* < .01), and * (*p* < .05).

^a^Rate per 10,000 population.

^b^Percent of tracts that were gentrifying, binary measure with no *t* test.

### Sex Work Arrests

Although 911 calls reporting sex work is our primary variable of interest to conceptualize vice complaints, we also include sex work arrests as an outcome for comparison. Arrests allow us to measure police control of sex work or where police most heavily enforce sex work regulations. We obtained Chicago Police Department (CPD) arrest data on all prostitution offenses from 2017 to 2020 through the Chicago Data Portal.^3^ The CPD provides these data to the city through its electronic reporting system. Each arrest record includes a crime incident (which can include multiple people arrested), the date, partially redacted block information, and a brief description of the arrest. There is no information on the attributes of the people arrested. We used the CPD’s description variable to confirm our inclusion of all the arrests the CPD labeled as prostitution, and over 96% of these arrests were for solicitation. We cleaned the arrest block data to midpointed addresses to obtain spatial coordinates through *ggmap*, and we merged these arrests to the 2020 census tract boundaries to generate tract level counts (like the 911 call data). On average, there were 601 sex work arrests per year from 2017 to 2020.^4^ We summed the four years of arrests per census tract to generate counts for modeling. During the four years, the 857 Chicago census tracts had an average of 2.8 (sd = 23.7) sex work arrests, with the tracts ranging from a minimum of zero to a maximum of 428 arrests. Not surprisingly, 911 calls and arrests are positively and moderately correlated at the tract level (Pearson correlation = 0.5). Our descriptive statistics highlight this overlap, and we test for differences across neighborhood variables by modeling arrests separately from 911 calls.

### Gentrification

As discussed above, scholarship has highlighted gentrifiers’ role in leveraging 911 to protect economic investments and quality-of-life in their neighborhoods. Our gentrification measure builds on the measure by Brazil and Portier (2021) to identify tracts that were gentrifying during the 2010s. We used the American Community Survey (ACS) five-year estimates (2006–2010) for our 2010 baseline and the five-year estimates (2015–2019) for our end of decade measures pulled from the *tidycensus* R package (Walker, 2023). The ACS did not release the 2020 one-year estimates due to data collection challenges during the COVID-19 pandemic (Walker, 2023), but we were able to crosswalk the 2015-2019 estimates to the 2020 census tract boundaries using *tidycensus* (Walker & Herman, 2021).

We first identified all census tracts that were gentrifiable in 2010 by noting tracts that were below the median tract value in (1) median rent, (2) median home value, (3) median household income, and (4) percent of the population with a Bachelor’s degree or higher.^5^ This first step removes advantaged neighborhoods that experience additional appreciation over time from the gentrification measure. Second, we calculated the percent change in these four variables from 2010 to 2019. We compared each tract’s percent changes to percent changes of the median tract values for each corresponding variable, and we coded tracts that had a higher percent change than the percent change in the medians. Lastly, we coded the gentrifiable tracts as gentrifying when three or four of the percent changes were higher than the median changes (Brazil & Portier, 2021). Based on this measure, 115 Chicago tracts (13.4%) gentrified during the 2010s.

### Residential and Commercial Land Use

Given much of the prior scholarship focuses on residents with some attention on quality-of-life policing near businesses, we are especially interested in the residential and commercial composition of neighborhoods reporting sex work to 911. While information on who called 911 (e.g., a business owner, customer, resident) is unavailable, we can measure commercial and residential land use composition at the tract level to identify the types of spaces where these calls originate. Similar to Boessen and Hipp (2018), we obtained data from the Chicago Metropolitan Agency for Planning’s (CMAP) Land Use Inventory dataset from 2015 for Northeastern Illinois (CMAP, 2015). CMAP measures land use as parcels, which are smaller units than tracts. Parcels are typically defined as a block, lot, or section of land delineated by boundaries or property lines. CMAP assigns each parcel a land use category, and our analysis focuses on two of these: residential and commercial. Residential land uses include single-family homes that are either free-standing or contained within multi-unit structures. Commercial land uses include shopping malls, regional and community retail centers, large-site retail businesses, smaller retail and trade services, mixed-use residential buildings, offices, hotels/motels, and entertainment centers. On average, there were approximately 222 parcels per census tract in Chicago. For each tract, we calculated the percentage of parcels that were residential and commercial. Chicago census tracts averaged 58% residential parcels and 12% commercial parcels. These two land use measures are not correlated (Pearson correlation = 0.1).

### Socioeconomic Disadvantage

Using the ACS five-year average (2015–2019), we created a measure of socioeconomic disadvantage using principal component analysis to index the following variables: (1) percent poverty, (2) percent unemployed, (3) percent single mother-headed households, and (4) median household income (in 2019 dollars). We mean centered and inversed the first component as our index, with higher values indicating greater socioeconomic disadvantage (see similar indices in Boessen & Hipp, 2018). The average socioeconomic disadvantage score for the 857 census tracts in Chicago was 0.0 (sd = 1.7, range -3.5–7.3).

### Percent Black Population

We measured the Black population as a percentage for each tract (mean = 34.2%, sd = 38.9%, range zero–100%).

### Analytic Strategy

**Figure 1.**
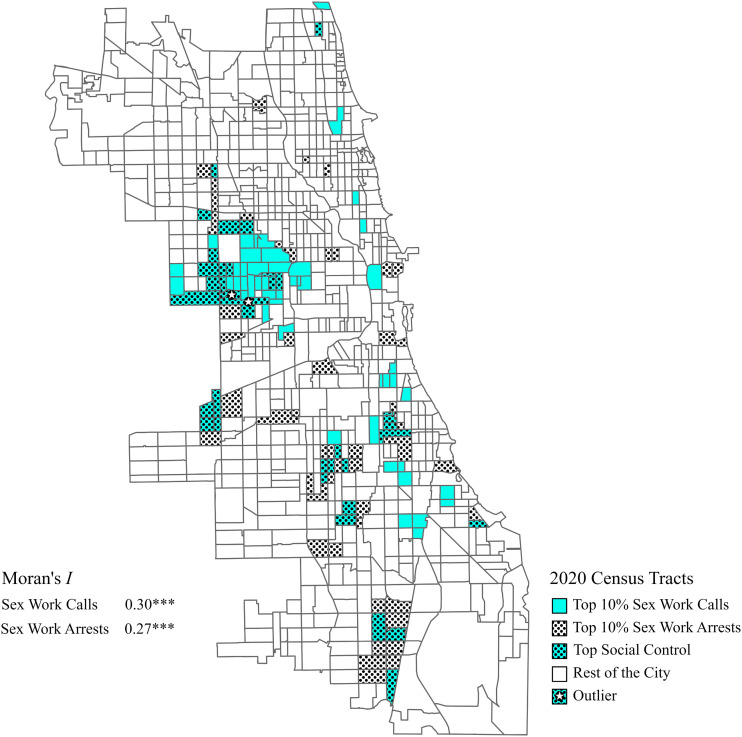
Map of Sex Work 911 Call and Arrest Rates by Census Tract in Chicago, 2017-2020.

Our dependent variable is the total count of 911 calls reporting sex work within a census tract from 2017 to 2020 (*n* = 857), and it sums the four years of calls per census tract to increase the variation across tracts and minimize annual fluctuation. Summing the four years of calls into a single time unit requires subsequent cross-sectional modeling. Even with the four-year totals, 911 calls for sex work are skewed with four-year counts per tract ranging from zero to over 2000 and with a standard deviation more than 1.5 times higher than the mean. Following Papachristos and colleagues’ (2018) comparison strategy of absolute homicide differences across Chicago neighborhoods, we identify the Chicago neighborhoods at the top 10% of sex work 911 call rates, the top 10% of sex work arrest rates, and the overlap of both forms of social control (top 4%), which we compare to the rest of the city (bottom 84%). Figure 1 presents a map showing the spatial distribution of the top 10% tracts and their overlap. To examine the spatial clustering that visually appears in the map, we conduct Moran’s *I* tests that assess the global correlation between a variable and its value among its contiguous neighbors (Weisburd et al., 2022).

Our multivariate analyses presented in Table 2 use negative binomial regression which is appropriate for analyzing overdispersed count data and common when modeling spatial crime data (Boessen & Hipp, 2018; Weisburd et al., 2022). Likelihood ratio tests confirm the overdispersion. We include tract level logged population as the offset in our models, which converts the dependent variable from counts to rates (Weisburd et al., 2022). We run variance inflation factor (VIF) post-estimation tests on all variables which were all below 2.3, well below the recommended cutoff of 5 (Weisburd et al., 2022, p. 110). We replicate our 911 call model on sex work arrests to test if the relationships for the neighborhood characteristics are unique to vice complaints or if the same relationships are present between neighborhood characteristics and arrests.^6^ Both models use clustered-robust standard errors to help adjust for overdispersion.

**Table 2.** Negative Binomial Regression Models Predicting Sex Work 911 Calls and Arrests per Census Tract in Chicago, 2017-2020.

	Model 1	Model 2
	911 Calls (SE)	Arrests (SE)
Gentrification^a^	1.26*	0.82
	(0.11)	(0.48)
% Residential land use	1.02***	0.98**
	(0.00)	(0.01)
% Commercial land use	1.07***	1.09***
	(0.00)	(0.02)
Socioeconomic disadvantage	1.36***	1.85***
	(0.03)	(0.16)
% Black	1.02***	1.02***
	(0.00)	(0.01)
Intercept	0.00	0.00
	(0.15)	(0.57)
*N*	857	857

*Notes*. Incidence rate ratios with standard errors in parentheses.

***p < .001, **p < .01, *p < .05. Tract level logged population is included as the offset in both models. SE = Clustered-robust standard errors.

^a^The reference category is non-gentrifying.

## Results

Table 1 presents averages and ranges across our measures by comparing neighborhoods with the top 10% of sex work 911 call rates (*n* = 86), the top 10% of sex work arrest rates (*n* = 86), the top social control neighborhoods that overlap in the highest rates of 911 calls and arrests (*n* = 37, 4% of tracts), and the rest of the city (*n* = 722, 84% of tracts). Two-tailed *t* tests confirm significant differences in the means of relevant variables between the top 10% neighborhoods and the rest of the city. The mean 911 call rate among the top 10% is 1,316.3 calls reporting sex work per 10,000 compared to the average rate of 149.1 for the rest of the city. The mean sex work arrest rate for the top 10% is 125.1 arrests per 10,000 population compared to the rate of 0.3 for the rest of the city. In Chicago’s neighborhoods with the top social control of sex work (calls and arrests), the ratio of arrest rates to call rates at the tract level shows that, on average, there is 1 arrest for every 6.6 calls targeting sex work per 10,000 population. However, in the rest of the city (bottom 84%), the ratio is 532.5 calls for every arrest, due to the very low arrest rate where calls are lower but still occurring. Table 1 also shows that the top social control tracts have significantly higher socioeconomic disadvantage and percent Black population compared to the rest of the city.

Figure 1 illustrates the spatial association between 911 calls and arrests across Chicago’s census tracts, indicating the top 10% call tracts in light blue, the top 10% arrest tracts in a dotted pattern, and the overlap of tracts with the top social control of sex work in both light blue and the dotted pattern. Figure 1 shows that although the top 10% calls and arrest tracts include islands of high urban social control of sex work from the northeast to the central business district, the clusters of social control occur in Chicago’s west and south sides. The Moran’s *I* values of 0.30 and 0.27 show only moderate spatial clustering citywide for sex work 911 calls and arrests, respectively. The hotspot for the social control of sex work is a stroll located at the southernmost edge of the West Garfield Park neighborhood. Two adjacent tracts in this neighborhood are outliers in our analysis (indicated by stars in Figure 1): tract 8430 had a 911 call rate of 8,583.6 and an arrest rate of 97.7 per 10,000 population, and tract 2610 had a 911 call rate of 6,028.1 and an arrest rate of 1,880.5 per 10,000 population. These two tracts are 93% and 92% Black and 45% and 30% in poverty, respectively. This well-known sex work hotspot generates public complaints from news coverage where residents describe sex work as “plaguing West Garfield Park for years,” and a previous Alderman stating “I’ve lived on the West Side of Chicago most of my life … and everybody knows to go to Madison Street to find a young lady… That’s not a reputation that I want the West Garfield community to have. We want to clean up that scourge” (Harris, 2021; Ostman, 2011).

Table 2 presents our multivariate analyses of negative binomial regression models with incident rate ratios (IRR) examining key neighborhood variables on the count of 911 calls for sex work in Model 1. Model 2 analyzes the same neighborhood variables predicting the count of arrests for sex work to identify any potential neighborhood differences between calls and arrests. We include tract level logged total population as an offset in both models, which controls for population and models our dependent variables as rates rather than counts.

Gentrifying neighborhoods are associated with a significant increase in sex work 911 calls relative to non-gentrifying neighborhoods (IRR = 1.26, *p* < .05, Model 1). However, gentrification does not have a significant relationship with sex work arrests relative to non-gentrifying neighborhoods (Model 2). Increases in the proportion of a tract’s residential land use is associated with a slight increase in 911 calls for sex work (IRR = 1.02, *p* < .001, Model 1), but increases in residential land use correspond with small decreases in sex work arrests (IRR = 0.98, *p* < .01, Model 2). Increases in the proportion of commercial land use correspond with small increases in the rate of 911 calls for sex work (IRR = 1.07, *p* < .001, Model 1), and we find this same relationship between commercial land use and sex work arrests (IRR = 1.09, *p* < .001, Model 2). Socioeconomic disadvantage has the largest positive impact on sex work 911 calls and arrests relative to the other variables in the models (IRR = 1.36, *p* < .001, Model 1; IRR = 1.85, *p* < .001, Model 2). Lastly, increases in the percent Black population also are associated with small increases in 911 calls and arrests for sex work (IRR = 1.02, *p* < .001, Model 1; IRR = 1.02, *p* < .001, Model 2).

## Discussion

Between 2017 and 2020, Chicago’s publics demonstrated significant efforts to resolve quality-of-life issues through 911 calls. Specifically, Chicagoans made over 65,000 calls during this period to complain about sex work, presumably in hopes of diverting illicit market activity away from the neighborhoods where they do business or live. We frame this study within theories of urban social control in which publics use 911 to activate police to manage perceived disorder and disorderly persons. The rates of 911 calls targeting sex work imply that many Chicagoans perceive sex work as a form of disorder requiring some legal intervention. We analyze these gendered vice complaints by neighborhood and relative to arrests to understand the frequency and spatiality of the urban social control of this form of disorder. Zeroing in on 911 calls reporting sex work considers spatial neighborhood processes that might be differently gendered compared to prior scholarship and adds a vantage point to previous research on the urban social control of disorder that largely overlooks gender.

Our first research question asks, where are 911 calls reporting sex work originating in Chicago? We find that sex work 911 calls spread across Chicago with only moderate spatial clustering. However, the tracts with the top 10% of sex work 911 calls concentrate in Chicago’s West Side—including the outlier tract in West Garfield Park with 2,109 calls (8,583.6 calls per 10,000 population). We situate the frequency and spatiality of 911 calls reporting sex work relative to arrests. Neighborhoods with the top social control of sex work (911 calls and arrests), on average, have a gap of 6.6 calls for every arrest per 10,000 population, whereas the rest of the city has a gap of 532.5 calls for every arrest per 10,000 population. Outside of the top 10% neighborhoods predominantly located in Chicago’s west and south sides, neighborhoods in the rest of the city still call 911 to report sex work, but these calls coincide with very low arrests rates. Police are not using crime control responses to manage sex work throughout much of Chicago, with the possible implication being that police deprioritize complaints about this gendered victimless crime in some parts of the city but not others.

Our second research question asks, what are the relationships between neighborhood characteristics and these gendered vice complaints? Our key statistical findings concerning this question center on the higher rates of 911 calls emanating from gentrifying, residential, commercial, socioeconomically disadvantaged, and higher percent Black neighborhoods. Socioeconomic disadvantage has the largest effect on 911 calls. We test these same neighborhoods characteristics on sex work arrests, and the positive relationships between percent commercial land use and percent Black population are similar for 911 calls and arrests. However, we find no relationship between gentrification and arrests, residential land use lowering arrests, and socioeconomic disadvantage having an even larger effect on arrests than 911 calls.

Prior research establishes that residents, especially gentrifiers, are frequent drivers of quality-of-life 911 calls. Our findings extend this claim to include reporting sex work. Interestingly, gentrifying neighborhoods do not have higher arrests for sex work, suggesting a mismatch between gentrifiers’ complaints and subsequent police responses in these neighborhoods. This finding compliments qualitative work by Oselin and colleagues (2022) showing that street-based sex workers prefer a popular stroll in a gentrifying neighborhood in Washington, DC, a location in which police did not make many arrests.

Building on the literature that finds that commercial districts use 911 to improve business fronts with dense ambient populations making calls, our statistical findings confirm that commercial areas also target sex work at the neighborhood level. Business owners and workers may feel that calling 911 is an effective way of diverting visible disorder away from their storefronts to avoid driving away potential customers. Our contribution to these literatures is that the motivations for urban social control from gentrifying and commercial neighborhoods include sex work as a differently gendered form of disorder. In addition to targeting homelessness or groups perceived to be gangs or drug dealers with calls demanding quality-of-life policing, we find that these complaints of disorderly persons also include sex workers and that these relationships are citywide.

Economically disadvantaged neighborhoods and Black neighborhoods experience disproportionate public crime and increased policing, and our study adds that this is also the case for the social control of sex work. Our statistical results show that the positive relationship of socioeconomic disadvantage is the strongest for 911 calls reporting sex work, and the percent Black population is associated with a small increase in 911 calls reporting sex work. Past research reveals that 911 calls disproportionately occur in racially segregated and disadvantaged communities, despite high levels of skepticism regarding the effectiveness of police in resolving disorder (Hagan et al., 2022). Black neighborhoods face additional complaints and police control of sex work even when controlling for socioeconomic disadvantage. We interpret these findings in relation to the established hierarchies within the sex industry. Street-based sex work requires fewer resources, is more accessible for workers, and often transpires in more dangerous settings compared to indoor sex markets. The visibility of outdoor sex work makes it especially vulnerable to 911 complaints and arrests in contrast to indoor sex work.

Prior research on the urban social control of disorder and quality-of-life policing has not primarily focused on the gendered illicit market of sex work. However, 911 calls reporting this gendered, victimless, and economic crime raise different orientations toward disorder. Rather than safety concerns or fear of crime, public concerns around sex work are replete with moral judgments (Shdaimah et al., 2014; Skogan, 1990) – tapping into criminalized dimensions of gender and sexuality. In other words, civilians are generally not afraid of women sex workers, but they condemn the behaviors and do not want to see sex work in their neighborhoods. Our contribution to these literatures includes our spatial findings of vice complaints spread across the city – not limited to certain neighborhoods, but concentrated within disadvantaged neighborhoods. Our findings that gentrifying, commercial, and socioeconomically disadvantaged neighborhoods are drivers of vice complaints show that different publics have similar motivations to control sex work as a form of disorder.

We acknowledge several limitations of this research. First, we do not have measures on why people call 911 to report sex work or any demographic information on the people they are reporting. Given the definition of this crime, we can surmise that the 911 callers are not victims of this illicit market. A Baltimore study found that quality-of-life concerns regarding “disgust and nuisance” were residents’ primary complaints about sex work in their communities (Shdaimah et al., 2014). Surveys or interviews would better unearth civilians’ motivations for 911 calls, including more specificity on how concerns pertaining to sex workers are gendered and the types of actions they incite. While illuminating, these approaches come at the cost of analyzing neighborhood differences. Our main data limitation is that earlier years of the 911 call data are unavailable despite our efforts to secure them by submitting multiple FOIA requests to several city offices. Ideally, we would have 911 call data as early as 2001 (which matches the CPD arrest data availability) in order to examine how calls have changed over time and what factors impact complaints. Lastly, the CPD arrest data do not capture any measure of “move-along” orders from police, which are common in complaint driven policing and force sex workers to temporarily relocate (Herring, 2019; Oselin et al., 2022).

## Conclusion and Policy Implications

In this study, we turn our focus to the gendered illicit market of criminalized sex work that disproportionately includes and targets cisgender and transgender women – and disproportionately poor women of color in street-based sex work. We draw on official 911 and arrest reports that do not fully capture the breadth or frequency of this illicit market but reveal unequal efforts to control it. Women typically participate in sex work out of economic necessity; yet, in doing so, they face social controls and other negative outcomes that compound their disadvantage. When neighborhood actors differently attempt to control disorder attributed to sex workers, they serve as collaborators in the policing and potential punishment of sex workers.

We see several policy implications based upon this study. The entanglement of the stigmatization and criminalization of sex work perpetuates public perceptions of sex workers as causing disorder and demanding police responses. Outside of the most controlled neighborhoods, the gap between 911 calls and arrests indicates that police are not actively responding to sex work complaints with arrests in much of Chicago. However, scholars caution that high rates of complaints have the potential to shift policing priorities and justify more punitive actions against sex workers (Rosentel et al., 2021). Moreover, complaints are not just emanating from residents. Our findings show that people in commercial districts are also calling 911 in relatively high numbers. This points to a type of quality-of-life policing in commercial landscapes with the potential force to divert sex workers away from business areas. It remains to be seen if and how this might further displace sex workers.

Our findings suggest that stigma continues to shroud this market, prompting complaints and police scrutiny that can further exacerbate harm for already disadvantaged people. Socioeconomically disadvantaged neighborhoods experience the most complaints and arrests of sex work, where residents may be most dependent on incomes generated from it. Black neighborhoods are also disproportionately targeted by these social controls. Sex workers are mobile and access customers in various ways, but visible outdoor sex workers in the poorest areas of Chicago experience disproportionate efforts to regulate and control their work. Most outdoor sex workers perform it due to intersectional inequalities linked to class, race/ethnicity, and gender statuses that disadvantage them in more prestigious, higher paying, and less stigmatized occupations.

While these 911 calls do not always result in arrests, they may still facilitate interactions with police that can produce other negative outcomes (e.g., harassment, violence, criminal records, forced relocation, or deteriorated health). Moreover, sustained public scrutiny has the potential to cause sex workers to engage in riskier, fleeting encounters with customers to avoid detection and complaints, such as shortened screening time of clients or operating in less visible areas where there are fewer safety resources. Policies that legally disentangle sex work from crime (e.g., decriminalization or legalization) offer greater protections for this marginalized population. By mapping 911 calls targeting sex work, we gain a better understanding of how they reflect spatial inequality in Chicago, how their concentration correlates with policing behavior, and which neighborhoods’ sex workers are most vulnerable to these gendered vice complaints.

## Notes

1. The three tracts with zero population are the former Robert Taylor Homes public housing (tract 3817), O’Hare Airport (tract 9800), and Midway Airport (tract 9801).

2. Annual totals of 911 calls reporting sex work are 20,074 in 2017, 18,122 in 2018, 15,589 in 2019, and 11,822 in 2020.

3. https://data.cityofchicago.org/Public-Safety/Crimes-2001-to-Present/ijzp-q8t2.

4. Annual totals of CPD sex work arrests are 730 in 2017, 718 in 2018, 680 in 2019, and 275 in 2020.

5. Three of the four census variables used in this measure required some cleaning (percent Bachelor’s degree or higher had no tracts with missing values). We adjusted the 2010 dollar-based variables (median rent, median home value, and median household income) for inflation to 2019 US dollars. We used linear interpolation within tracts over time for tracts with missing values in median rent and median household income. Our linear interpolations included census data from 1990, 2000, 2010, 2015, and 2019. Median rent had 4 tracts in 2010 and 7 tracts in 2019 with missing values, and median household income had 1 tract in 2019 with a missing value. We used spatial interpolation to replace missing values in median home value based on averaging the home values of contiguous tracts due to the high fluctuations within tracts over time. Median home value had 2 tracts in 2010 and 8 tracts in 2019 with missing values.

6. Due to the overdispersed distribution of our count variables, we are unable to fit spatial autocorrelation models. Following the example in Minkoff (2016), we test a spatial lag term based on the contingency matrix’s weights for the dependent variable. The spatial lag is significant and positive, but with small effect sizes (IRR = 1.01). This suggests that there is some spatial clustering in our dependent variables at the tract level, but all of the relationships presented in Table 2 hold.
